# Abiraterone-Induced Secondary Hypertension: Two Wrongs Don’t Make a Right

**DOI:** 10.7759/cureus.60299

**Published:** 2024-05-14

**Authors:** Achilleas Betsikos, Eleni Paschou, Virginia Geladari, Stauroula Magaliou, Nikolaos Sabanis

**Affiliations:** 1 First Department of Internal Medicine, General Hospital of Trikala, Trikala, GRC; 2 General Practice and Family Medicine, 10th Local Medical Unit of Giannouli, Larisa, GRC; 3 Nephrology Department, General Hospital of Trikala, Trikala, GRC

**Keywords:** hypokalemia, mineralocorticoid excess syndrome, prostate cancer (pca), secondary hypertension, abiraterone

## Abstract

Abiraterone, an inhibitor of both 17α-hydroxylase and 17,20-lyase, is considered a novel, state-of-the-art, life-prolonging therapy in the urologists’ arsenal when treating prostate cancer. Despite its efficacy, it is linked with an increased risk of cardiovascular adverse effects. Herein, we report a case in which the administration of abiraterone resulted in a full-blown syndrome of apparent mineralocorticoid excess despite the concomitant administration of prednisolone; that is, secondary hypertension, hypokalemia, metabolic alkalosis, as well as elevated levels of adrenocorticotropic hormone (ACTH).

## Introduction

Abiraterone, an inhibitor of both 17α-hydroxylase and 17,20-lyase, is an oral targeted antineoplastic medication for the treatment of metastatic castration-resistant prostate cancer alternative to traditional cytotoxic chemotherapies since 2011 [1]. Despite its efficacy and long-term safety, abiraterone is not without serious adverse reactions attributable to its mechanism of action, namely the inhibition of androgen and cortisol biosynthesis [2].

The latter causes a rise in adrenocorticotropic hormone (ACTH) levels, resulting in an increase in mineralocorticoids that is associated with serious cardiovascular side effects, including hypertension, hypokalemia, and fluid overload. This is the classical clinical triad, also known as mineralocorticoid excess syndrome (MES) [3]. Thus, the addition of a glucocorticoid to abiraterone is considered imperative to prevent the occurrence and severity of MES by reducing excess ACTH. However, the exact dose of prednisone or dexamethasone that is needed remains elusive, given that the occurrence of MES is reported even with the co-administration of a glucocorticoid in approximately 15% of patients. In the same context, another field of uncertainty is the optimal therapeutic strategy for MES, which includes the use of amiloride in combination with hydrochlorothiazide rather than the administration of an aldosterone blocker such as spironolactone or eplerenone that may activate androgen receptors, resulting in further castration-resistant prostate cancer [4]. 

Herein, abiraterone-induced endocrine hypertension is part of mineralocorticoid excess syndrome and represents another cause of secondary hypertension in an ever-growing list of medications. Taking into account the above, in this article, we describe a case of secondary and resistant hypertension in a patient receiving abiraterone for castrate-resistant metastatic prostate cancer and briefly discuss the difficulties and challenges encountered concerning the prevention, recognition, and management of MES, the most common and on-target side effect of abiraterone, especially in patients of advanced age with serious underlying comorbidities.

## Case presentation

An 81-year-old man was referred to this hospital by his family doctor with signs and symptoms of acute heart failure. At the presentation, the patient complained about dyspnea. He was alert, anxious, and mildly diaphoretic, with prominent pedal edemas. On auscultation, there were crackles on all lung fields. The blood pressure was 195/65 mmHg, the heart rate was 73 beats per minute, and the oxygen saturation was 92% while the patient was breathing ambient air. Supplemental oxygen was delivered through a venturi mask. A urinary catheter was inserted. Intravenous furosemide and nitroglycerine were administered, and a systolic blood pressure target of 130 mmHg was achieved. This patient’s breathlessness quickly alleviated. Afterward, a computed tomography of the chest was ordered to exclude other possible causes of dyspnea besides acute heart failure. Laboratory tests revealed marked hypokalemia of 2.2 mg/dl, mild hypernatremia of 145 mg/dl, anemia, and metabolic alkalosis (pH=7.52). Other laboratory parameters are presented in Table 1. Subsequently, the patient was transferred to the internal medicine department.

**Table 1:** Laboratory examination WBC: white blood cells; HT: hematocrit; HB: hemoglubin; SGOT: serum glutamic-oxaloacetic transaminase; SGPT: serum glutamic pyruvic transaminase; LDH: lactate acid dehydrogenase; CPK: creatine phosphokinase; N/A: non applicable

	Day 1	Day 2	Day 3	Day 4	Day 5	Normal Ranges
WBC*	4660	4800	4810	4550	4100	4-10.8 10^3^/μL
Ht	29.7	33.9	34.4	34.2	32.9	37.7-47.9 %
Hb	9.9	11.2	11.2	11.4	10.9	11.8-17.8 g/dL
Platelets	201.000	182.000	193.000	185.000	181.000	150-350 10^3^/μL
Glucose	104	91	98	90	115	75-115 mg/dL
Urea	56	32	38	42	40	10-50 mg/dL
Creatinine	1.01	0.84	0.79	0.69	0.78	0.40-1.10 mg/dL
Sodium	141	145	145	145	144	136-143 mg/dL
Potassium	2.47	2.20	2.52	2.74	2.84	3.5-5.1 mg/dL
Magnesium	2.51	N/A	2.53	N/A	2.34	1.6-2.6mg/dL
SGOT*	37	N/A	37	N/A	35	5-40 IU/L
SGPT*	20	N/A	22	N/A	22	10-37 IU/L
LDH*	437	N/A	296	N/A	220	135-225 IU/L
CPK*	161	N/A	102	N/A	49	24-190 IU/L
C-reactive protein	0.56	4.04	4.35	3.59	2.53	<0.7 mg/dL

Apart from heart failure with reduced ejection fraction (HFrEF), other medical history included hypothyroidism, atrial fibrillation, hypertension, and castrate-resistant metastatic prostate cancer. Medications included levothyroxine, omeprazole, rivaroxaban, metoprolol tartrate, amlodipine, perindropril, and furosemide. For prostate cancer treatment, the patient has received a combination of dutasteride and tamsulosin for the past two years. Three weeks before the presentation, abiraterone plus prednisone were added to this scheme. There were no reports of allergies, alcohol use, or tobacco use.

Despite the initial control of hypertension on admission, systolic blood pressure measurements were consistently over 165 mmHg. Moreover, the severe hypokalemia persisted despite the high doses of intravenous potassium chloride over 24 hours. No electrocardiographic manifestations of hypokalemia were noted. Based on the above, 50 mg of eplerenone was added to the anti-hypertensive regimen, but to no avail, so it was discontinued. On the fifth day of hospitalization, both hypertension and hypokalemia were still present; hence this warranted an investigation of secondary causes of hypertension. It must be noted that the oncology service suggested that the anti-neoplastic regimen with abiraterone should be continued during hospitalization.

Taking into consideration the recent initiation of the anti-androgen abiraterone and its unique mechanism of action, ACTH levels were ordered. As expected, the ACTH levels were almost five times above the normal range (256 pg/ml; normal values are 10-60 pg/ml). This particular result guided the administration of 4 mg of dexamethasone. In the next three days, ACTH levels, sodium, and potassium levels normalized while the blood pressure was on target (<140 mmHg). The patient was subsequently discharged with a recommendation to continue receiving 4 mg of dexamethasone along with the prescribed abiraterone. One month after this hospitalization, the patient was re-evaluated at the nephrology department of our hospital. In coordination with the oncology service, abiraterone was replaced by a combination of leuproreline/enzalutamide, and steroid weaning was decided since long-term steroid use bears significant risk.

## Discussion

**Figure 1:**
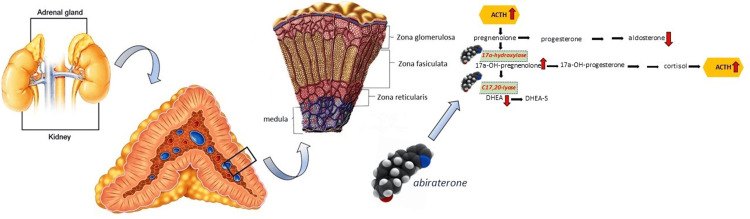
Mineralocorticoid excess syndrome Steroid synthesis in the adrenal gland. Zona glomerulosa is responsible for the production of the mineralocorticoid aldosterone. The enzyme CYP17A is expressed in zona fasciculata and zona reticularis, resulting in the production of cortisol and dehydroepiandrosterone-sulfate (DHEA-S), respectively. The administration of abiraterone inhibits both hydroxylase and lyase activity, resulting in mineralocorticoid excess syndrome. ACTH: adrenocorticotropic hormone (created by E. Paschou and N. Sabanis)

Steroid synthesis in the adrenal gland occurs in the gland’s three zones. The zona glomerulosa is responsible for the production of the mineralocorticoid aldosterone. The enzyme CYP17A is expressed in the zona fasciculata and the zona reticularis, resulting in the final production of cortisol and dehydroepiandrosterone-sulfate (DHEA-S), respectively. In each zone, this particular enzyme functions in a different fashion. In the former, there is hydroxylase activity, while in the latter, there is lyase activity [5]. The administration of abiraterone inhibits both of these enzymatic activities. Therefore, the production of cortisol and DHEA-S is disrupted, and this metabolic pathway is driven towards the production of 11-deoxy-corticosterone and corticosterone. This marked decrease in the levels of cortisol activates a feedback stimulation loop by which secondary elevation of the ACTH levels occurs and results in even higher levels of both 11-deoxy-corticosterone and corticosterone. This effect suppresses the renin-angiotensin system so that aldosterone production is also inhibited [6]. Despite this, corticosterone has an affinity for the mineralocorticoid receptor that is comparable to aldosterone, and given its 40-fold increase, a syndrome resembling apparent mineralocorticoid excess ensues [7,8] (Figure 1). To remediate this, abiraterone is always prescribed in combination with a corticosteroid [9].

Secondary causes of hypertension are reported in 5-15% of hypertensive people [10]. Drug-induced secondary hypertension is even rarer. A fairly long list of medications and other substances that may raise blood pressure exists and keeps expanding [11] (Table 2). According to the European Society of Hypertension, suspicion of secondary hypertension should be raised when treating hypertensive people with specific characteristics, including resistant hypertension, severe hypertension, a hypertensive emergency, biochemical and/or clinical evidence suggestive of an endocrine cause, and acute worsening hypertension in patients of advanced age with previously longstanding stable normotension [12]. In our case, resistant hypertension in a previously normotensive individual was noted.

**Table 2:** Causes of secondary hypertension

Causes	
Renal parenchymal disease	Diabetic kidney disease
	Polycystic kidney disease
	Glomerular diseases
Renovascular disease	Atherosclerotic renovascular disease
	Fibromuscular dysplasia
Endocrine causes	Primary aldosteronism
	Pheochromocytoma
	Cushing’s syndrome
	Thyroid disease (hyperthyroidism or hypothyroidism)
	Hyperparathyroidism
Obstructive sleep apnea	
Other causes	Coarctation of the aorta
Drugs	Non-steroidal anti-inflammatory drugs: ibuprofen, celecoxib, naproxen
	Hormonal therapies: oral contraceptive pill, testosterone
	Illicit drugs: cocaine, amphetamines, ecstasy
	Herbal preparation: ginseng, liquorice, St John’s wort, efedra, ma huang
	Decongestants: phenylephrine, pseudoephedrine
	Immunosuppressants: corticosteroids, calcineurin inhibitors
	Caffeine
	Nicotine
	Psychiatric drugs: venlafaxine, bupropion, clozapine, tricyclic antidepressants, fluoxetine, carbamazepine, lithium, monoamine oxidase inhibitors
	Antineoplastic agents: vascular endothelial growth factor inhibitors, tyrosine kinase inhibitors, abiraterone, alkylating agents
	Erythropoietin stimulating agents
	Alcohol

The combination of resistant hypertension, marked hypokalemia, and metabolic alkalosis constitutes the triad of aldosteronism. This patient met all three criteria, so we also considered, in the differential diagnosis, primary aldosteronism, renin-secreting tumors, and renal artery stenosis. The initial presentation in the emergency department resembled the infamous Pickering syndrome [13], i.e., flushing pulmonary edema due to bilateral renal artery stenosis. However, this patient had normal kidney function. To emphasize this point even further, renal function tests were normal despite the prescribed angiotensin-converting enzyme (ACE) inhibitor. Furthermore, the CT scan of the abdomen did not reveal any adrenal gland or kidney abnormalities, and the aldosterone levels were within the normal range. These findings render the diagnosis of these situations highly implausible. Of note, rare genetic forms of hypertension were not taken into account based on this patient’s age. In our patient, an acquired enzymatic disorder secondary to abiraterone seemed much more likely. Even though prednisone was already administered, we did show that it didn’t suffice to break the ACTH feedback loop. Hence, we proceeded with the replacement of prednisone with relatively high doses of dexamethasone and monitored the ACTH levels until normalization.

To continue the commentary on this case, major pitfalls must be emphasized. The administration of abiraterone is linked with well-established cardiovascular risk, as shown in the pharmacovigilance study performed by Cone et al. [14]. Therefore, we suggest that it would be preferable to prescribe a different class of anti-androgen in this case, since this patient was afflicted by HFrEF as well as atrial fibrillation. The decision to include abiraterone in the inpatient drug regimen constituted without a doubt another “wrongdoing” with dual repercussions. On the one hand, there was an ongoing exposure of this patient to the cardiac adverse effects of abiraterone, all the more so since he presented to the ER with the clinical picture of hypertensive acute heart failure, a major adverse cardiovascular event. On a continuum with the aforementioned, in our opinion, this warrants immediate discontinuation of the drug. On the other hand, it rendered the correction of the potassium and blood pressure levels even harder.

Last but not the least, it is important to comment on the decision to use mineralocorticoid receptor antagonists, such as eplerenone. Although this option seems rational, given the HFrEF as well as the hypokalemia, it comes with a significant risk of interaction with abiraterone’s mechanism of action. Authors in the field propose the use of amiloride as an alternative potassium-sparing diuretic [15]. Unfortunately, in Greece, amiloride is not available as a single drug pill and only comes in combination with other diuretics.

## Conclusions

Novel drug therapies are driving a shifting landscape in established knowledge, and modern medicine is becoming more and more complex. To the best of our knowledge, this is the first reported case in a clinical setting where the anti-neoplastic drug, abiraterone, induced a secondary form of hypertension in a fashion resembling mineralocorticoid excess. The successful management of this case covered a multitude of faults along the way. Given that abiraterone heightens cardiovascular risk, this case underscores the importance of exercising caution when prescribing abiraterone to patients who have cardiovascular comorbidities.

## References

[1] Lai LY, Oerline MK, Caram ME, Tsao PA, Kaufman SR, Hollenbeck BK, Shahinian VB (2022). Risk of metabolic and cardiovascular adverse events with abiraterone or enzalutamide among men with advanced prostate cancer. J Natl Cancer Inst.

[2] Attard G, Reid AH, Auchus RJ (2012). Clinical and biochemical consequences of CYP17A1 inhibition with abiraterone given with and without exogenous glucocorticoids in castrate men with advanced prostate cancer. J Clin Endocrinol Metab.

[3] Fizazi K, Tran N, Fein L (2019). Abiraterone acetate plus prednisone in patients with newly diagnosed high-risk metastatic castration-sensitive prostate cancer (LATITUDE): final overall survival analysis of a randomised, double-blind, phase 3 trial. Lancet Oncology.

[4] Decamps S, Lis R, Ekanayake P (2023). Abiraterone-induced endocrinopathies. JCEM Case Rep.

[5] Zobniw CM, Causebrook A, Fong MK (2014). Clinical use of abiraterone in the treatment of metastatic castration-resistant prostate cancer. Res Rep Urol.

[6] Ang JE, Olmos D, de Bono JS (2009). CYP17 blockade by abiraterone: further evidence for frequent continued hormone-dependence in castration-resistant prostate cancer. Br J Cancer.

[7] Gomez-Sanchez E, Gomez-Sanchez CE (2014). The multifaceted mineralocorticoid receptor. Compr Physiol.

[8] Fuller PJ, Yao Y, Yang J, Young MJ (2012). Mechanisms of ligand specificity of the mineralocorticoid receptor. J Endocrinol.

[9] Fizazi K, Tran N, Fein L (2017). Abiraterone plus prednisone in metastatic, castration-sensitive prostate cancer. N Engl J Med.

[10] Rimoldi SF, Scherrer U, Messerli FH (2014). Secondary arterial hypertension: when, who, and how to screen?. Eur Heart J.

[11] Grossman A, Messerli FH, Grossman E (2015). Drug induced hypertension--an unappreciated cause of secondary hypertension. Eur J Pharmacol.

[12] Williams B, Mancia G, Spiering W (2018). 2018 ESC/ESH Guidelines for the management of arterial hypertension. Eur Heart J.

[13] T G Pickering, L Herman, R B Devereux (1988). Recurrent pulmonary oedema in hypertension due to bilateral renal artery stenosis: treatment by angioplasty or surgical revascularisation. Lancet.

[14] Cone EB, Reese S, Marchese M, Nabi J, McKay RR, Kilbridge KL, Trinh QD (2021). Cardiovascular toxicities associated with abiraterone compared to enzalutamide- a pharmacovigilance study. EClinicalMedicine.

[15] Bedussi F, Galli D, Fragni M (2017). Amiloride is effective in the management of abiraterone-induced mineralocorticoid excess syndrome without interfering with its antineoplastic activity. Pharmacology.

